# Impact of rs11024102 *PLEKHA7*, rs3753841 *COL11A1 s*ingle *n*ucleotide polymorphisms, and serum levels of oxidative stress markers on the risk of primary angle-closure glaucoma in Egyptians

**DOI:** 10.1186/s43141-022-00400-w

**Published:** 2022-08-29

**Authors:** Marwa Aswa, Hazem Helmy, Shahira Noweir, Somaia Ismail, AlShaimaa Taha, Azza Atef

**Affiliations:** 1 Department of Biochemistry, Faculty of Science, Ain Shams University, Abbassia, Cairo, 11566 Egypt; 2 Department of Glaucoma and Optic Nerve Disease, Research Institute of Ophthalmology, Giza, Egypt; 3 Department of Human Genetics, Research Institute of Ophthalmology, Giza, Egypt; 4 Department of Medical Molecular Genetics, Division of Human Genetics and Genome Research, National Research Center, Giza, Egypt

**Keywords:** Polymorphism, Association, Malondialdehyde, Advanced glycation-end product, Protein carbonyl, Risk factors, Predictors, Intraocular pressure

## Abstract

**Background:**

Primary angle-closure glaucoma (PACG) is one of the major causes of blindness in the Middle East with genetic loci and systemic oxidative stress as potential risk factors. The current case-control study aimed to investigate the associations of rs11024102 in Pleckstrin homology domain-containing family A member 7 (*PLEKHA7*), rs3753841 in collagen 11 A1 (*COL11A1*), and the systemic oxidative stress markers with PACG in Egyptian patients. Thirty-five control subjects and 64 PACG patients were enrolled in this study. The polymorphisms in *PLEKHA7* and *COL11A1* were analyzed using quantitative PCR, and their associations were statistically tested with PACG at *homozygous, heterozygous, dominant, and recessive genetic models*. The levels of malondialdehyde (MDA), advanced glycation-end product (AOPP), protein carbonyl (PC), and ischemia modified albumin (IMA) were quantitated colorimetrically, and their associations with PACG were analyzed statistically. The associations of MDA, AOPP, PC, and IMA with elevated intraocular pressure (IOP) were statistically tested.

**Results:**

Neither significant difference in the genotype distribution nor allele frequency of *PLEKHA7* 11024102 T>C (*p* = 0.425 and 0.517, respectively) and *COL11A1* rs3753841 *G>A* (*p* = 0.600 and 0.473, respectively) were recorded under any of the tested genetic models. Either rs11024102 *PLEKHA7* or rs3753841 *COL11A1* was not significantly (*p* > 0.025 after Bonferroni correction) associated with an increased risk of PACG in Egyptians*. *Egyptian patients with PACG showed significant elevations in the serum levels of MDA, AOPP, and PC either in patients with or without cases with diabetes mellites, hypertension, coronary vascular diseases, and smoking. Serum levels of MDA, AOPP, and PC were significantly associated with PACG in Egyptians (*p* < 0.013 after Bonferroni correction). However, MDA and PC only showed significant associations with the elevation in the IOP (p = 0.007 and 0.045, respectively) in PACG patients.

**Conclusion:**

*Both* rs11024102 and rs3753841 could not be considered as potential gene-dependent risk factors for PACG pathogenesis in Egyptians. On the other hand, serum levels of MDA, AOPP, and PC might be considered risk factors for PACG. Moreover, MDA and PC could serve as good predictors for the elevation of the IOP in PACG disease.

## Background

Primary angle-closure glaucoma (PACG), a neurodegenerative disease, is responsible for about 50% of glaucoma blindness in African-American patients [1]. In 2020, 20 million subjects (40–80 years old) are suffering from PACG worldwide and this number is expected to increase to 34 million people by 2040 [2, 3]. The prevalence of PACG is the highest in Asia (0.73%), including the Middle East compared to Caucasians and Africans [4].

In PACG, the mechanical obstruction of the trabecular meshwork (TM) is by either apposition of the peripheral iris to TM or a synechia closed-angle resulting in elevated intraocular pressure (IOP) [5]. The rise of IOP causes eye pain, blurry vision, redness, headache, rainbow-colored rings (“haloes”) around the light, and nausea or vomiting [6]. The risk factors for PACG include age, female gender, ocular biometric features, and ethnicity (e.g., African and Chinese) [7].

Multifactorial causes are implicated in the pathogenesis of PACG including genetic factors and oxidative stress. The genetic susceptibility for PACG is still under investigation in different populations. Several PACG loci and genes have been recently identified by genome-wide association studies (GWAS), which may shed light on the molecular mechanisms of PACG [8, 9]. Among these genes, the human Pleckstrin homology domain-containing family A member 7 (*PLEKHA7*) and collagen 11 A1 (*COL11A1*) were addressed.

The human *PLEKHA7* gene is located on chromosome11p15.1 and encodes an adherens junction protein [10] which is required for organizing the epithelial architecture [11], tissue homeostasis [12], and controlling the fluid flow through the inner wall of the Schlemm’s canal [13]. It was reported that the genetic variants of *PLEKHA7* gene are implicated in the pathogenesis of PACG by affecting the fluid dynamics [8]. The *COL11A1* gene is located on chromosome 1p21, consists of 68 exons, and encodes collagen type XI [14]. Different *COL11A1* gene variants are associated with type II Stickler and Marshall syndromes [15–17] which are congenital conditions that include high myopia and blindness from retinal detachment [18].

Numerous research work evaluated the association of single-nucleotide polymorphisms (SNPs) rs11024102 in *PLEKHA7* and rs3753841 in *COL11A1* with PACG in different populations all over the world. In Nepalese and Chinese individuals, *PLEKHA7* rs11024102 is found to be associated with PACG (*P*=0.039 and 0.0024, respectively) [19, 20]. On the other hand, no statistically significant association was reported between *PLEKHA7* rs11024102 and PACG in Australians (*P*= 0.411) [19] or South Indians (*P*= 0.213) [21]. *COL11A1* rs3753841 is significantly associated with PACG in Australians (*P* = 0.017) [19]. On the contrary, no statistically significant association was reported between *COL11A1* rs3753841 and PACG in the Nepalese, Chinese, and South Indian subjects (*P*= 0.308, 0.054, and 0.127, respectively) [19–21].

Sacca et al. [22] reported a significant positive correlation between PACG and oxidative stress in which oxidative stress induces human TM DNA damage, visual field damage, and an elevation in IOP. Li et al. [23] documented the implication of oxidative stress in the development of PACG and visual field loss by reducing the antioxidants in association with increasing malondialdehyde. Recently, oxidative stress induced by PACG was reported to elevate IOP and cause peroxidation of fatty acids and reduce their levels in plasma resulting in the production of MDA that can damage retinal cells by cytotoxicity and proliferation inhibition [24].

According to our knowledge, the present study aimed to evaluate for the first time the association between rs11024102 in *PLEKHA7* and rs3753841 in *COL11A1* with the pathogenesis of PACG in Egyptians. In addition, the current study investigated the association between systemic oxidative stress on the one side and PACG and the rise in IOP on the other side, to evaluate the probability of oxidative stress as a risk factor for PACG in Egyptians.

## Methods

### Sample size calculation

The estimation of sample size was performed using the *χ*^2^ test (both variables are dichotomous). It was assumed that there are no associations between the studied variables and PACG in Egyptians as a null hypothesis, while associations between the studied variables and PACG in Egyptians were assumed as the alternative hypothesis. Power of study = 80%, *α* error (two-sided) = 0.05, *β* error = 0.2, and effect size = odds ratio (OR) of 3.

It was postulated that P1 = the proportion of patients expected to have the studied variables in the PACG group. The proportion of control subjects having the studied variables (P2) and the authors found to be about 0.2. The value of P1 was estimated from the equation of P1 = (OR*P2)/[(1-P2) + (OR*P2)] and found to equal 0.43. Based on this estimation, a total of 64 PACG patients were enrolled in this study.

### Patients

The current case-control study was approved by the ethical committee of the Research Institute of Ophthalmology (RIO), Giza, Egypt. All procedures were conducted under the Declaration of Helsinki. Written informed consent was obtained from all control subjects and PACG patients before participating in the study. A total of 64 PACG patients were recruited from the glaucoma unit of the Research Institute of Ophthalmology (RIO), Giza, Egypt. These patients were diagnosed by an expert glaucoma consultant (HH). On the other hand, 35 control subjects matched in age and gender were recruited from the refractive clinic of RIO during their annual refractive check-up.

All patients underwent full ophthalmological examination that included best-corrected visual acuity (BCVA) by Landolt C optotype chart converted to log MAR scale, intraocular pressure (IOP) measurement by Goldman applanation tonometer (GAT) (Haggy–sterit AT 90, Swiss-made), three values were assessed, and the mean value was documented. Gonioscopy with Zeiss 4 mirror goniolens (model OPSDG, ocular instruments, USA) was performed, and the grade of angle was signed by Shaffer scale for angle grading. Anterior chamber depth was documented according to the van Herrick technique. Fundus examination was performed by 90 D lens aided with slit-lamp biomicroscopy. Angle examination was performed by anterior segment OCT (AS-OCT, Optvue, Fermont, CA, USA) and B scan ultrasonography (Sonomed UBM 35MHZ transducer), and angle width in degrees was documented. This examination included also an evaluation of anterior chamber depth (ACD) and crystalline lens. ACD was documented by an A-scan ultrasonography examination (Sonomed, USA).

#### Inclusion and exclusion criteria

Inclusion criteria included PACG subjects with eyes having narrow angles and elevated IOP (IOP>21 mm Hg) as well as subjects receiving glaucoma medications. In addition, both PACG and control subjects with no major systemic diseases (e.g., autoimmune disease or cancer) were included in the study. On the other hand, patients with secondary angle-closure glaucoma, congenital glaucoma, trauma, uveitis, iris, or angle neovascularization as well as patients with any ocular disease that may alter the anatomy of the anterior segment as a tumor, iris, or ciliary body mass or surgery were excluded.

### Sample collection

A volume of 5-ml venous blood samples was collected from each subject. Two milliliters of blood was collected on tubes containing ethylenediaminetetraacetic acid (EDTA) anticoagulant and used for DNA isolation and the analysis of the selected genetic polymorphisms. The remaining 3 ml of the blood were collected and left for 2 h at room temperature for complete clotting followed by centrifugation at 3000 rpm to obtain serum which was then collected, aliquoted, and stored at −20°C until analyzing the selected markers of oxidative stress.

### DNA extraction and genotyping

The total genomic DNA was extracted from the blood of all subjects using a Quick-DNA^TM^ Miniprep kit (Zymo Research, USA) according to the manufacturer’s protocol. The extracted DNA concentrations were measured using NanoDrop (Nano Drop 2000/2000c, Thermo Scientific™). Analyses for the rs11024102 and rs3753841 polymorphisms were accomplished by using TaqMan® SNP Genotyping Assay kit (Applied Biosystems, Thermo Fisher Scientific, USA). This analysis was done in a Step One™ real-time PCR (Applied Biosystems, Thermo Fisher Scientific, USA) with a total reaction volume of 20 μl containing 3 μl of the extracted DNA (20 ng), 10 μl TaqMan® Genotyping Master Mix, 0.5 μl TaqMan® SNP Genotyping Assay (TaqMan probes), and 6.5 μl nuclease-free water. The thermal conditions of real-time PCR consisted of denaturation at 95°C for 10 mins of initial cycle, denaturation at 95°C for 15 s (40 cycles), and annealing/extension at 60°C for 1 min (40 cycles).

### Analysis of oxidative stress markers

#### Malondialdehyde assay

Malondialdehyde (MDA) was quantified in serum as thiobarbituric acid-reacting substance (TBARS) production as described by Esterbauer and Cheeseman [25]. The absorbance of the samples was determined at 532 nm. The amount of TBARS was calculated by comparison with authentic malondialdehyde.

#### Advanced oxidation protein products assay

The level of advanced oxidation protein products (AOPP) was assayed in serum according to the method of Witko-Sarsat et al. [26] using standard chloramine-T solution (Sigma, St Louis, MO, USA) ranging from 0 to 100 mmol/L. The levels of AOPP were expressed as mmol/L of chloramine-T equivalents.

#### Protein carbonyl assay

The levels of protein carbonyl (PC) were estimated in serum by the spectrophotometric assay described by [27]. The absorbance of each sample was spectrophotometrically measured at 370 nm. The level of protein carbonyl (nmol/ml) was calculated using ε_M_ = 22,000.

#### Ischemia-modified albumin assay

The level of ischemia-modified albumin (IMA) was measured in serum as reduced cobalt-to albumin binding capacity using the colorimetric assay described by Bar-Or et al. [28] using 0.1% cobalt chloride and dithiothreitol (Sigma, Sigma-Aldrich Corporation, St. Louis, MO). The absorbance of each sample was measured at 470 nm using a spectrophotometer. IMA was calculated from the difference between the absorbance of samples measured with and without dithiothreitol. The results were expressed as absorbance units (ABSU).

### Statistical analysis

All statistical analyses were performed using the Statistical Package for Social Science version 20 for Windows (SPSS software package, Chicago, USA). The distribution of data was statistically determined using the Kolmogorov-Smirnov test with Lilliefors significance correction. The categorical variables are expressed as frequencies (percentages) while the quantitative variables are expressed as mean ± standard error (SE). The quantitative variables in the two studied groups were statistically analyzed using the independent *t* test. *χ*^2^ test was used to evaluate the Hardy–Weinberg equilibrium (HWE) and differences in allele frequencies for each SNP between the PACG and control groups.

Binary logistic regression analyses were used to investigate the strength of the association between the *PLEKHA7* and *COL11A1* gene polymorphisms and the susceptibility to PACG using various genetic models. The association was measured by the odds ratio (OR) with a 95% confidence interval (CI) adjusted for sex and age. The strength of association of the oxidative stress markers was measured in all cases and cases without DM, hypertension, CVD, and smoking by OR with 95% CI adjusted for sex and age. For association analyses using binary logistic regression, the statistical significance thresholds were set to *p* < 0.025 and *p* <0.013 after Bonferroni correction for the studied gene variants and markers of oxidative stress, respectively. Multiple linear regression analysis was used to assess the association of the independent variables (MDA, AOPP, PC, and IMA concentrations) relative to the elevated IOP average (dependent variable). The regression models were constructed using the “enter” analysis. All *p* values were 2-sided, and a *p* value < 0.05 was considered statistically significant.

## Results

### Demographics, clinical, and ocular characteristics

This case-control study comprised 64 PACG patients aged 59.77±8.10 years old including 56.2% males and 43.8% females. The control subjects were chosen to be matched in age (56.71±6.79 years old) with PACG patients. Forty percent of males and 60% of females were enrolled in this study as control subjects.

Table 1 summarizes the demographics, clinical, and ocular characteristics of subjects in PACG and control groups. There was no statistical difference (*p*>0.05) in the SBP, DBP, pulse rate, the presence of *diabetes mellites*, hypertension, smoking, and CVD between PACG and control subjects. The IOP average was significantly elevated in the PACG group (41.18%, *p*<0.0001), compared to the control group. Of PACG patients, 34 were under topical glaucoma medications while 18 patients were exposed to intraocular surgery at least 2 months before collecting the blood samples.

**Table 1:** Demographics, clinical, and ocular characteristics of subjects in controls and PACG groups

	Control group (*n*=35)	PACG group (*n*=64)	*T* value/*χ*^2^ value	*P* value
Age (years)	56.71±6.79	59.77±8.10	−1.89	0.061
Gender *n* (%)				
Male	14 (40)	36 (56.2)	2.390	0.122
Female	21 (60)	28 (43.8)		
SBP (mm Hg)	126.49±14.18	132.75±18.03	−1.78	0.079
DBP (mm Hg)	81.06±6.40	81.47±10.88	−0.205	0.838
Pulse rate (BPM)	74.86±.6.94	78.20±9.64	−1.811	0.073
IOP average (mm Hg)	15.47±0.31	21.84±0.71	−6.47	<0.0001
DM (yes/no)	4/31	12/52	0.895	0.344
Topical glaucoma medications (yes/no)	0/35	60/4	58.617	<0.0001
Hypertension (yes/no)	6/29	21/43	2.801	0.094
Smoking (yes/no)	2/33	4/60	0.011	0.915
Intraocular surgery (yes/no)	0/35	18/46	12.03	0.001
CVD (yes/no)	1/34	7/57	1.989	0.158

Data are expressed as mean ± SE for quantitative variables and frequencies (percentages) for categorical variables

*PACG* primary closure angle glaucoma, *SBP* systolic blood pressure, *DBP* diastolic blood pressure, *BMP* beats per minute, *IOP* intraocular pressure, *DM* diabetes mellitus, *CVD* cardiovascular diseases

The mean difference is significant at *p*<0.05

#### Genotypic distribution and allelic frequencies

The distribution of genotypes and the frequencies of alleles of *PLEKHA7* and *COL11A1* genes among control and PACG subjects are summarized in Table 2. The genotyping distribution of *PLEKHA7* 11024102 T>C and *COL11A1* rs3753841 G>A polymorphism was concordant with HWE in the control and PACG groups (p > 0.05). For* PLEKHA7* 11024102 T>C, there was no statistically significant difference in the genotype distribution and allele frequency in the PACG group, compared to the control group (*p* = 0.425 and 0.517, respectively). Also, *COL11A1* rs3753841 *G>A, *PACG patients showed no statistically significant difference in the genotype distribution and allele frequency (*p* = 0.600 and 0.473, respectively), compared to the control group.

**Table 2:** Genotype distribution and allele frequency of the studied genes in control and PACG groups

		Control group (*n*=35)	PACG group (*n*=64)	*χ*^2^ value	*P* value
*PLEKHA7* 11024102 T>C					
Genotype distribution, *n* (%)	TT	27 (77.1)	54 (84.4)	1.711	0.425
	TC	8 (22.9)	9 (14.1)		
	CC	0 (0)	1 (1.66)		
	*p-*HWE	0.196	0.256		
Allele frequency (%)	T	88.6	91.4	0.419	0.517
	C	11.4	8.6		
*COL11A1* rs3753841 *G>A*					
Genotype distribution, *n* (%)	GG	9 (25.7)	11 (17.2)	1.023	0.600
	GA	18 (51.4)	37 (57.8)		
	AA	8 (22.9)	16 (25)		
	*p-*HWE	0.773	0.103		
Allele frequency (%)	G	51.4	46.1	0.516	0.473
	A	48.6	53.9		

Data are expressed as frequencies (percentage)

*PLEKHA7* Pleckstrin homology domain-containing family A member 7*, COL11A1* collagen 11 A1, *p-HWE p* value of Hardy–Weinberg equilibrium

#### *PLEKHA7* 11024102 T>C and *COL11A1* rs3753841 G>A polymorphisms and PACG association

Binary logistic regression analyses were used after adjustment for age and gender to evaluate the association of *PLEKHA7* 11024102 T>C and *COL11A1* rs3753841 G>A polymorphisms with PACG risk using homozygous, heterozygous, dominant, and recessive genetic models (Table 3). Both *PLEKHA7* 11024102 T>C SNP and *COL11A1* rs3753841 G>A SNP showed no significant association was recorded with PACG risk under any of the tested genetic models (*p*>0.025 and *p*>0.025, respectively), after Bonferroni correction.

**Table 3:** *PLEKHA7* and *COL11A1* variants and PACG risk according to genetic association models using binary logistic regression

	PACG vs. control	
	^#^Adjusted OR (95% CI)	*P* value
*PLEKHA7* rs11024102 T>C		
Homozygous model (CC versus TT)	7.28 × 10^8^	1
Heterozygous model (TC versus TT)	0.44 (0.15–1.36)	0.156
Dominant model (TT/TC versus TT)	0.50 (0.17–1.49)	0.211
Recessive model (CC versus TC/TT)	7.59 × 10^8^	1
Major allele (T)	0.60 (0.22–1.64)	0.319
*COL11A1* rs3753841 *G>A*		
Homozygous model (AA versus GG)	1.18 (0.49–6.76)	0.375
Heterozygous model (GA versus GG)	1.54 (0.51–4.66)	0.45
Dominant model (GG/GA versus GG)	1.56 (0.55–4.43)	0.401
Recessive model (AA versus GA/GG)	1.00 (0.37–2.72)	1
Major allele (G)	1.16 (0.63–2.22)	0.635

*PLEKHA7* Pleckstrin homology domain-containing family A member 7, *COL11A1* collagen 11 A1, *OR* odds ratio, *95% CI* 95% confidence interval

^#^Adjusted for sex and age. *n*= 35 for the control group and 64 for PACG patients

*P* < 0.025 was considered significant after the Bonferroni correction

#### Markers of systemic oxidative stress

**Fig. 1:**
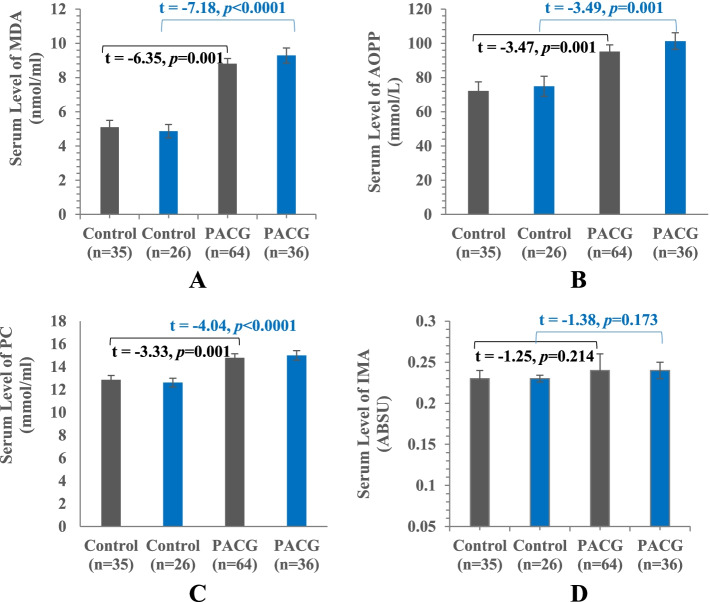
Levels of systemic oxidative stress markers in serum of all control (*n*=35) and PACG subjects (*n*=64) as well as after excluding subjects with DM, hypertension, CVD, and smoking (*n*=26 for control, *n* = 36 for PACG). **A** MDA, **B** AOPP, **C** PC, and **D** IMA. Data are expressed as mean ± SE. PACG, primary angle-closure glaucoma; MDA, malondialdehyde; AOPP, advanced oxidation protein products; PC, protein carbonyl; IMA, ischemia-modified albumin; ABSU, absorbance units. The mean difference is significant at *p*<0.05

Figure 1 illustrates the incidence of oxidative stress in all PACG patients in the form of significant elevations in the serum levels of MDA (72.75%, *p* = 0.001), AOPP (31.82%, *p* = 0.001), and protein carbonyl (15.08%, *p* = 0.001), compared to the control group. However, no significant elevations were observed in the serum level of IMA in PACG patients, compared to the control subjects.

To examine if the oxidative stress incident in the PACG group is only due to the disease not due to the presence of *diabetes mellitus*, hypertension, CVD, and smoking, the analyses were re-assayed after excluding these cases (Fig. 1). The independent *t* tests proved the same results obtained in Table 4 with variations in the change percentage. Significant elevations in MDA (90.76%, *p <* 0.0001), AOPP (35.27%, *p* = 0.001), and PC (18.95%, *p* < 0.0001) were observed, compared to the control group. Furthermore, no significant elevations were observed in the serum level of IMA.

**Table 4:** Association between *markers of systemic oxidative stress* and PACG risk using binary logistic regression

	PACG vs. control	
	^#^Adjusted OR (95% CI)	*P* value
MDA (nmol/ml)^a^	1.70 (1.35–2.14)	<0.0001
MDA (nmol/ml)^b^	2.50 (1.56–4.01)	<0.0001
AOPP (mmol/L)^a^	1.03 (1.01–1.05)	0.001
AOPP (mmol/L)^b^	1.04 (1.01–1.07)	0.005
PC (nmol/ml)^a^	1.43 (1.15–1.78)	0.001
PC (nmol/ml)^b^	1.74 (1.25–2.44)	0.001
IMA (ABSU)^a^	89.22 (0.004–1.87 × 10^6^)	0.376
IMA (ABSU)^b^	1.51 × 10^3^ (0.01–4.77 × 10^8^)	0.257

*PACG* primary angle-closure glaucoma, *OR* odds ratio, *95% CI* 95% confidence interval, *MDA* malondialdehyde, *AOPP* advanced oxidation protein products, *PC* protein carbonyl, *IMA* ischemia modified albumin, *ABSU* absorbance units

^#^Adjusted for sex and age

^a^All subjects (*n*=35 for control subjects, *n*=64 for PACG patients)

^b^Control and PACG subjects after excluding subjects with DM, hypertension, CVD, and smoking (*n*= 26 for the control group and 36 for PACG patients)

*P* < 0.013 was considered significant after the Bonferroni correction

#### Markers of systemic oxidative stress and PACG association

To examine the probability of MDA, AOPP, protein carbonyl, and IMA as risk factors for PACG, binary logistic regression analyses were used to evaluate their associations with PACG with and without cases of DM, hypertension, CVD, and smoking (Table 4) after adjusting for age and gender using Bonferroni correction of *p* < 0.013. Significant associations between MDA (OR = 1.70, 95% CI = 1.35–2.14, *p* < 0.0001), AOPP (OR = 1.03, 95% CI = 1.01–1.05, *p* = 0.001), and PC (OR = 1.43, 95% CI = 1.15–1.78, *p* = 0.001) were reported in all PACG subjects. Similarly, significant associations of the serum levels of MDA (OR = 2.50, 95% CI = 1.56–4.01, *p* < 0.0001), AOPP (OR = 1.04, 95% CI = 1.01–1.07, *p* = 0.005), and PC (OR = 1.74, 95% CI = 1.25–2.44, *p* = 0.001) were reported when excluding DM, hypertension, CVD, and smoking cases. No significant associations (*p* > 0.013) of IMA with PACG were recorded among all PACG subjects as well as after excluding DM, hypertension, CVD, and smoking cases, after Bonferroni correction.

#### Markers of systemic oxidative stress and prediction of IOP elevation

To explore if the systemic oxidative stress markers are probable predictors for the elevation of IOP average in the PACG group, multiple linear regression analyses were performed among all PACG subjects as well as among PACG without DM, hypertension, CVD, and smoking cases (Table 5). Among all PACG patients, MDA and PC were found to be the only predictor for elevating the IOP average with a 0.29 standardized coefficient (*β*) and *t* value of 2.86 at *p* value = 0.005. On contrary, both MDA (*β* = 0.34, *t* = 2.80 at *p* = 0.007) and PC (*β* = 0.25, *t* = 2.05 at *p* = 0.045) were found to be significant predictors for elevating IOP average after excluding DM, hypertension, CVD, and smoking cases.

**Table 5:** Multiple linear regression analysis for the association of systemic oxidative stress markers with the IOP average

Dependent variable	Predictors	*R*	*r*^2^	*F*	*P* value^*^	*β*	*t*	*P* value	VIF
IOP average^a^	MDA (nmol/ml)	0.40	0.13	4.49	0.002	0.29	2.86	0.005	1.17
	AOPP (mmol/L)					−0.07	−0.66	0.510	1.18
	PC (nmol/ml)					0.15	1.53	0.129	1.08
	IMA (ABSU)					0.18	1.84	0.07	1.09
IOP average^b^	MDA (nmol/ml)	0.50	0.25	4.83	0.002	0.34	2.80	0.007	1.11
	AOPP (mmol/L)					−0.06	−0.51	0.612	1.08
	PC (nmol/ml)					0.25	2.05	0.045	1.11
	IMA (ABSU)					0.21	1.78	0.08	1.04

*IOP* intraocular pressure, *β* standardized coefficient, *VIF* variance inflation factor, *MDA* malondialdehyde, *AOPP* advanced oxidation protein products, *IMA* ischemia-modified albumin, *ABSU* absorbance units

^*^P value obtained from ANOVA table

^a^All subjects (*n*=35 for control subjects, *n*=64 for PACG patients), ^b^Control and PACG subjects after excluding subjects with DM, hypertension, CVD, and smoking (*n*= 26 for the control group and 36 for PACG patients)

*P* < 0.05 was considered significant

#### Effect of topical glaucoma medications on systemic oxidative stress and IOP average

After excluding PACG cases with DM, hypertension, CVD, and smoking (*n*=34 patients), the effect of topical glaucoma medications was statistically estimated on the IOP average and the markers of systemic oxidative stress in PACG patients who took (*n*=32) or not (*n*=2) these medications (Table 6). There was no statistical difference in the elevated IOP average and the serum levels of AOPP and PC among PACG patients using or not these medications (*p*>0.05). Excitingly, serum levels of MDA were significantly higher in PACG patients under these medications (74.68%, *p* = 0.033) than those under no topical medications. Although there was no statistical change in the serum level of IMA in PACG patients in comparison with the control groups (Fig. 1), but also IMA level was found to be statistically lowered in PACG patients under topical glaucoma medications (46.67%, *p* = 0.006), compared to PACG patients who have been under no medications.

**Table 6:** The IOP average as well as the systemic *oxidative stress* in PACG patients using or not using the topical glaucoma medications

	Intraocular medications (no), (*n* = 2)	Intraocular medications (yes), (n= 34)	*T* value	*P* value
IOP average (mm Hg)	21.25±3.75	21.38±0.96	−0.03	0.974
MDA (nmol/ml)	5.45±0.65	9.52±0.44	−2.33	0.033
AOPP (mmol/L)	92.60±12.37	101.83±5.08	−0.43	0.668
PC (nmol/ml)	14.47±0.34	15.03±0.45	−0.30	0.769
IMA (ABSU)	0.45±0.01	0.24±0.01	2.91	0.006

Data are expressed as mean ± SE

*PACG* primary angle-closure glaucoma, *MDA* malondialdehyde, *AOPP* advanced oxidation protein products, *PC* protein carbonyl, *IMA* ischemia-modified albumin, *ABSU* absorbance units

The statistical analysis was performed on PACG patients after excluding subjects with DM, hypertension, CVD, and smoking

*P* < 0.05 was considered significant

## Discussion

More than 17 million people worldwide suffer from PACG. The prevalence of PACG varies widely across different ages, sex, and population geographic variation. Asian, gender (female), and age are risk factors for PACG [29]. PACG is a significant public health concern because of its severity which can elevate the risk of blindness. Several genes and loci have been identified by the genetic studies and found to be associated with PACG across different ethnicities. Eight genetic loci in different genes have been reported by GWAS investigations to be significantly associated with susceptibility to the pathogenesis of PACG in various populations [30].

The most potentially susceptible genes for PACG are *PLEKHA7* and *COL11A1* which can affect the angle structure because of their role in collagen synthesis and function [9]. *PLEKHA7* is expressed in the TM, iris, and cornea, and it encodes a protein that maintains and stabilizes the epithelial and endothelial adherens junctions. Eyes with PACG have abnormal anatomic biometric characteristics which hinder the aqueous humor outflow facility [10, 31]. In the present study, no significant association between rs11024102 *PLEKHA7* and PACG in Egyptian patients was found using any of the tested genetic models.

The *COL11A1* is expressed primarily in the articular cartilage and the ocular vitreous, and it codes for one of the two 𝛼-chains of type XI collagen, minor fibril-forming collagen, which controls fibril growth, diameter, and assembly of major collagens in the interstitial extracellular matrix. Mutations in *COL11A1* cause Stickler syndrome, Marshall syndrome, and Stickler-like syndrome which are manifested by abnormal collagen in the sclera with axial myopia [8, 18]. The hyperopic eyes are risk factors for PACG. In addition, *COL11A1* is also expressed in TM cells which may enhance IOP directly through aqueous outflow [32–34]. In the current study, no significant association between rs3753841 *COL11A1* and PACG in Egyptian patients was found using any of the tested genetic models.

The associations of the studied SNPs and PACG vary from one population to another and also vary in the same population. In Saudi Arabia, no association was recorded between rs11024102 *PLEKHA7* (OR = 1.53, *p* value = 0.140) and PACG while a significant association was found between rs3753841 *COL11A1* (OR = 1.4, *p* value = 0.026) [8]. Shi et al. [35] reported no significant associations between rs11024102 *PLEKHA7* (OR = 1.13, 95% CI = 0.88–1.44, *p* value = 0.346) and rs3753841 *COL11A1* (OR = 0.88, 95% CI = 0.68–1.15, *p* value = 0.369) SNPs in Chinese population with PACG. In Han Chinese population, significant association of rs11024102 *PLEKHA7* (OR = 1.15, 95% CI = 1.01–1.30, *p* value = 0.038) and PACG were recorded while no association of rs3753841 *COL11A1* (OR = 1.14, 95% CI = 0.99–1.31, *p* value = 0.062) was found [20].

Another study revealed no associations of rs11024102 *PLEKHA7* (OR = 1.02, 95% CI = 0.88-1.19, *p* value = 0.780) and rs3753841 *COL11A1* (OR = 1.15, 95% CI = 0.98–1.35, *p* value = 0.090) SNPs with PACG in Chinese and rs11024102 *PLEKHA7* (OR = 1.06, 95% CI = 0.85–1.33, *p* value = 0.60) and rs3753841 *COL11A1* (OR = 1.14, 95% CI = 0.93–1.41, *p* value = 0.21) in Indians [36]. In the study of Wan et al. [34], no association was reported between rs11024102 *PLEKHA7* (OR = 1.128, 95% CI = 0.647–1.965, *p* value = 0.671) in Chinese patients while significant association were recorded with rs3753841 *COL11A1* (OR = 1.8866, 95% CI = 1.083–3.426, *p* value = 0.036). A recent study by Thangavelu et al. [37] found no association between rs11024102 (*PLEKHA7*) SNP with PACG progression in Malays patients.

A better understanding of the serum levels of oxidative stress markers and their possible role in PACG may be clinically useful in the management of the disease. Previous studies have evaluated the levels of oxidative stress markers in the aqueous humor [38, 39] and peripheral blood [40, 41] of patients with glaucoma suggesting the implication of oxidative stress in the pathogenesis of glaucoma. MDA, AOPP, protein carbonyl, and IMA are well-known markers in the pathologic molecular process in oxidative stress.

Malondialdehyde is the end product of peroxidized polyunsaturated fatty acid decomposition [42]. Biomarkers of protein oxidation (AOPP and protein carbonyl) are often applied when a battery of markers of oxidative stress status is being studied. AOPP is synthesized by the action of chlorinated oxidants during oxidative stress [43–45]. The measurement of carbonyl groups is a good estimator for the extent of oxidative damage to proteins. Elevation of serum levels of PC is present in glaucoma patients, compared to the control subjects [46]. Chang et al. [40] considered IMA a highly sensitive biomarker for PACG. In acute ischemic conditions, IMA is formed due to the reduced binding capacity of albumin to transition metals resulting in a metabolic variant of the protein [47]. The current study confirmed the previously mentioned studies since MDA, AOPP, and PC were significantly elevated in the serum of Egyptian patients with PACG regardless of the presence of hypertension, *diabetes mellitus*, CVD, and smoking. These elevations were significantly associated with the pathogenesis of PACG. These findings are in agreement with the studies of Chang et al. [40] and Li et al. [23].

Oxidative stress was documented to be a risk factor in the development of PACG by damaging the nucleic acids, proteins, and lipids causing TM cells and retinal ganglion cell (RGC) damage [48]. Moreover, oxidative stress stimulates neuroinflammation by releasing cytokines [49] leading to the death of RGC by activating receptor-mediated inflammation signaling [50] enhancing the antigen presentation [51] and causing complementing dysregulation [50, 52]. Furthermore, RGCs are sensitive to PACG-generated oxidative stress [53], which is mainly produced by the mitochondria to activate apoptosis by activating caspase-3 and releasing cytochrome C [54]. All of these accelerate the progression of glaucoma by increasing RGC death and decreasing their proliferation.

Elevated IOP is considered the most important risk factor in the progression of PACG [55]. IOP is raised in PACG due to the obstruction in the aqueous humor outflow through the TM and Schlemm’s canal [56]. The elevated IOP induces changes in the mitochondria that generate ROS production and consequently accelerates the formation of oxidative adducts which is associated with retinal damage [57, 58]. This occurs via abnormal cristae loss, cytochrome C release, adenosine triphosphate reduction [59–61], retinal nitrite and lipid peroxidation elevation, retinal antioxidants reduction, glutaminergic neurotoxicity [62–64], retinal vascular dysregulation, TM damage, and retinal endothelial dysfunction [65, 66].

In Egyptians, the significant associations of both MDA and PC with the elevation in IOP average in PACG patients did not vary either in the presence of DM, hypertension, CVD, and smoking or in their absence. PACG therapy mainly focuses on reducing IOP and preventing or slowing down PACG progression. However, their therapeutic efficacy is not sufficient and disease progression continues despite treatment in most PACG patients. These suggest that there are factors other than high IOP that could be a therapeutic target for PACG; the incidence of systemic oxidative stress is one of these factors [67].

The current study showed a comparison between PACG patients using or not the topical glaucoma medications. Using or not these medications showed elevated IOP average as well as serum AOPP and PC levels. However, using these medications showed an elevation in the serum level of MDA as well as a reduction in the serum IMA level, compared to those who did not take these medications. IMA is a marker of ischemia-induced injury which is one of the causes of PACG progression [40]. Hence, targeting IMA with topical medications may be important to slow down the progression of PACG via controlling ischemia. But this was not enough for curing PACG due to the elevations in the other oxidative stress markers as well as the elevation in the IOP average. All of these results suggest that reducing MDA, AOPP, and PC is important in treating PACG in parallel with lowering the elevated IOP average. The limitations in this comparison in the present study are the number of PACG patients who did not use the topical medications and the collection of eye humor aqueous samples from PACG patients.

## Conclusions

In conclusion*,* *PLEKHA7* *rs11024102 T>C and* *COL11A1* *rs3753841* G>A SNPs could not be considered risk factors for PACG in Egyptians. On the other hand, systemic oxidative stress (the elevating serum levels of MDA, AOPP, and protein carbonyl) was found to be a probable risk factor for PACG. Of these markers, only serum levels of MDA and PC were considered significant predictors for the elevation in the IOP average. Future studies involving evaluating other SNPs in *PLEKHA7* and *COL11A1* genes are warranted to identify possible risk factors for PACG pathogenesis in Egyptians with increasing the sample size. Moreover, further studies should be performed in Egyptian patients with PACG to explore if there are any SNPs in the antioxidant enzymes that could explain the present elevations in the markers of oxidative stress.

## Data Availability

The datasets used and/or analyzed during the current study are available from the corresponding author on reasonable request.

## Abbreviations

ABSU: Absorbance units
AOPP: Advanced glycation-end products
CI: Confidence interval
GWAS: Genome-wide association studies
HWE: Hardy–Weinberg equilibrium
IMA: Ischemia-modified albumin
IOP: Intraocular pressure
MDA: Malondialdehyde
OR: Odds ratio
PACG: Primary angle-closure glaucoma
PC: Protein carbonyl
PLEKHA7: Pleckstrin homology domain-containing family A member 7
COL11A1: Collagen 11 A1
RGC: Retinal ganglion cells
SNPs: Single-nucleotide polymorphisms
TM: Trabecular meshwork
VIF: Variance inflation factor
β: Standardized coefficient
